# Effects of Band-Pull Walking Using a Portable Device on Cardiorespiratory and Neuromuscular Responses in Healthy Young Adults

**DOI:** 10.3390/sports14040130

**Published:** 2026-03-25

**Authors:** Ryota Tsuchiya, Hisashi Naito, Shuichi Machida, Keisuke Takamiya, Koji Sugiyama

**Affiliations:** 1 Graduate School of Health and Sports Science, Juntendo University, Inzai 270-1695, Japan; hnaitou@juntendo.ac.jp (H.N.); machidas@juntendo.ac.jp (S.M.); 2 Graduate School of Education, Shizuoka University, Shizuoka 422-8529, Japan; takamiya.keisuke.17@shizuoka.ac.jp

**Keywords:** walking with loaded upper extremities, elastic resistance, treadmill walking, energy expenditure, electromyography, perceived exertion

## Abstract

Upper-limb involvement during walking increases metabolic demand compared with normal walking (WK); however, methods such as Nordic walking or hand-held weights require technical skills or may increase mechanical load. This study examined the effects of upper-limb-resisted walking using a novel portable elastic resistance device (band-pull walking; BPW) on cardiorespiratory and neuromuscular responses in healthy young adults. Fourteen healthy young adults performed BPW and WK on a treadmill at 60, 80, and 100 m·min^−1^ in a randomized crossover design. Upper-limb resistance was individually standardized using triceps brachii activity (8% maximum voluntary contraction). Surface electromyography (EMG) of upper- and lower-limb muscles, oxygen uptake, heart rate, and perceived exertion were recorded. BPW significantly increased triceps brachii, biceps brachii, and deltoid muscle activity compared with WK at all or higher speeds (*p* < 0.05), whereas vastus lateralis and gastrocnemius lateralis activity remained unchanged. Metabolic equivalents and heart rate were higher during BPW across all speeds (*p* < 0.01), with increases of 8–12%. Upper-limb and whole-body perceived exertion were elevated, whereas lower-limb perceived exertion remained stable. These findings suggest that BPW was associated with increases in upper-limb muscle activation and metabolic demand, whereas no detectable increases were observed in vastus lateralis or gastrocnemius lateralis EMG activity or perceived lower-limb exertion under the present experimental conditions.

## 1. Introduction

From the perspectives of preventing lifestyle-related diseases and extending healthy life expectancy, promoting physical activity that can be easily incorporated into daily life is essential. Normal walking (WK) is widely recommended as a representative aerobic exercise because it does not require either special skills or equipment and can be performed safely and continuously [1,2]. Exercise intensity during WK is typically adjusted by increasing walking speed or incline; however, in older adults, individuals with obesity, those with knee pain or musculoskeletal disorders such as osteoarthritis, or populations with low exercise tolerance, increasing intensity through speed or incline may be limited due to reduced walking capacity or restrictions on weight-bearing activities [3,4,5,6,7]. Therefore, exercise modalities that increase metabolic demand without increasing lower-limb loading may represent useful alternatives in exercise prescription. Practical applicability depends on the characteristics of the target population, including pain, joint function, and exercise tolerance, highlighting the need for a stepwise evaluation based on fundamental physiological verification.

Upper-limb-involved walking, wherein the upper limbs are actively recruited during walking, represents a promising approach. Nordic walking, a representative example, has consistently been shown to increase oxygen uptake ($\overset{・}{V}$O_2_), energy expenditure, and heart rate (HR) compared with WK at the same speed [8,9,10,11,12]. Electromyographic studies indicate that Nordic walking enhances the activity of upper-limb muscles, including the triceps brachii (TB), deltoid (DM), and biceps brachii (BB), whereas lower-limb muscle activity shows small or inconsistent changes depending on the muscles and experimental conditions [13,14,15]. The physiological effects of Nordic walking are influenced by the proficiency in pole handling and poling style, which can generate inter-individual variability in practical settings [13,16]. Dumbbell walking also increases metabolic demand; however, holding heavy loads can increase postural control demands, induce fatigue, and impose additional stress on the joints and muscles [17,18,19]. Therefore, a method that preserves the advantages of upper-limb-involved walking while applying external loads in a standardized and reproducible manner is warranted. Arm swing during walking is not merely an auxiliary movement, but a critical biomechanical component in regulating trunk rotation and angular momentum [20,21]. Experimental studies have shown that restricting or modifying arm movement affects gait energetics and neuromuscular coordination, suggesting that upper-limb dynamics play an important role in locomotor control [22].

Elastic resistance training using bands or tubes has gained attention as a low-cost and portable exercise modality that can be implemented without specialized equipment in a wide range of settings [23]. Previous studies have reported that elastic resistance training can produce strength gains comparable to those achieved with conventional resistance exercises using weight machines or free weights [23]. A distinctive feature of elastic tubing is that the tensile force increases with elongation, resulting in variable resistance throughout the range of motion [24,25]. Consequently, resistance progressively increases toward the end of the movement, allowing muscles to generate force while being elongated and maintaining tension across the full movement trajectory [26]. Furthermore, elastic band-based interventions have demonstrated beneficial effects on muscle strength and functional performance in older adults, including individuals with sarcopenia, as well as in rehabilitation populations [27]. Collectively, these findings suggest that elastic resistance training can induce neuromuscular adaptations comparable to traditional resistance training while offering advantages in portability and practical applicability. Based on these characteristics, attempts have been made to apply elastic resistance during dynamic functional movements such as walking. Previous research indicates that walking with resistance applied to arm swing using elastic tubes increases upper-limb muscle activity and metabolic responses [28,29,30]. However, in these studies, one end of the elastic tube was fixed to an external structure such as a wall or stationary frame, thereby restricting movement space and limiting ecological validity. Consequently, these models were not designed for self-contained, free-living walking conditions.

In contrast, band-pull walking (BPW) was specifically developed as a self-contained portable walking modality. The elastic tubes are mounted on the trunk and connected to both hands, eliminating the need for external fixation and enabling resistance to be generated dynamically in synchrony with natural arm swing. Thus, BPW integrates elastic resistance directly into the gait cycle rather than superimposing externally anchored loading. Furthermore, whereas previous elastic-resisted walking approaches primarily defined resistance by the tube properties alone, the present study standardized resistance using an individualized percentage maximum voluntary isometric contraction (%MVC) reference of the TB. The TB is a primary elbow extensor, and elbow extension during the backward phase of arm swing is mechanically consistent with elastic tube elongation. TB activity can be assessed using standardized surface electromyography (EMG) procedures and TB has been commonly evaluated in studies of upper-limb-involved walking, including Nordic walking [8,14]. This physiologically based load prescription reduces inter-individual variability and enhances reproducibility. Accordingly, BPW differs conceptually and methodologically from prior elastic-resisted walking models in terms of portability, biomechanical integration with gait, and standardized load prescription.

Several translational challenges must be addressed to establish an upper-limb-resisted walking modality that is both standardized and practically applicable. First, differences in body size, upper-limb length, and movement patterns may lead to substantial inter-individual variability in resistance magnitude and metabolic responses, complicating load standardization. Second, existing elastic-resisted walking models require external fixation, thereby limiting implementation in community or outdoor settings. Third, certain upper-limb-involved walking modalities, such as Nordic walking, depend in part on technical proficiency, which may affect the reproducibility of exercise effects and uniformity of intervention delivery. Therefore, a walking modality that minimizes these sources of variability, eliminates environmental constraints, and provides a physiologically based method for load prescription would represent a meaningful translational advancement. BPW was developed with these considerations in mind. However, no previous study has comprehensively examined the physiological responses to BPW through the concurrent evaluation of muscle activity, metabolic and circulatory responses, and localized rating of perceived exertion (RPE) under identical walking speed and incline conditions. Uncertainty persists regarding whether BPW increases upper-limb muscle activity, and whether such increases elevate metabolic load expressed as metabolic equivalents (METs) and circulatory responses reflected by HR; additionally, it is unclear whether lower-limb muscle activity and lower-limb subjective exertion remain within acceptable ranges without excessive elevation.

The present study was designed as an exploratory proof-of-concept investigation to characterize the physiological features of BPW in healthy young adults under controlled laboratory conditions. Given the preliminary nature of this investigation, the purpose of this study was to examine, using an intra-subject crossover design, whether BPW increased upper-limb muscle activity and metabolic and circulatory responses compared with WK, without excessive increases in lower-limb muscle activity or lower-limb perceived exertion. The hypothesis proposed that BPW would increase the activity of TB, DM and BB, accompanied by higher METs and HR, whereas the activity of vastus lateralis (VL) and gastrocnemius lateralis (GL) and the lower-limb RPE would remain comparable to that of WK.

## 2. Materials and Methods

### 2.1. Participants

Fourteen healthy adults (nine men and five women) who engaged in regular physical activity participated in this study. Among men, the mean age was 22.9 ± 2.3 years, height was 172.2 ± 5.4 cm, body mass was 70.4 ± 10.7 kg, and body fat percentage was 12.4 ± 4.1%. Among women, the mean age was 21.2 ± 1.1 years, height was 161.5 ± 5.7 cm, body mass was 51.3 ± 7.0 kg, and body fat percentage was 18.8 ± 6.3% (Table 1). All participants reported no history of neurological, cardiovascular, or metabolic diseases and were not taking medications known to affect exercise capacity or physical function. Additionally, no participant reported a current smoking habit or a history of upper- or lower-limb injury within the past 6 months. Sample size determination was guided by previous studies involving walking and upper-limb-involved exercise paradigms [31,32]. The inclusion of both men and women was intended to reflect the general population of healthy young adults. Resistance intensity was prescribed relative to individual %MVC, thereby standardizing relative neuromuscular load across participants. Because the primary objective was to examine intra-subject responses across walking conditions, and the study was not specifically designed to evaluate sex-related effects, analyses were conducted on the pooled sample.

All participants were fully informed of the purpose, procedures, and the anticipated risks and benefits of the study, and written informed consent was obtained. Participants were instructed to refrain from strenuous exercise and alcohol consumption on the day before measurement, to sleep normally, and to avoid caffeine intake on the day of the measurement.

### 2.2. Study Design and Band-Pull Walking Setup

This study adopted a randomized crossover design to compare physiological responses between walking using a portable elastic tube device (BPW) and WK within the same participants. The order of BPW and WK was randomized across participants. In contrast, the walking speed was fixed at 60, 80, and 100 m·min^−1^ in this order for all participants to ensure safety and to increase exercise intensity in a stepwise manner. Therefore, although the speed factor may involve a time-dependent effect, the primary comparison in this study focused on differences between conditions (BPW vs. WK) at the same speed, and speed was treated as a repeated factor. Walking was performed on a treadmill with a 3% incline, with each speed maintained for 3 min, resulting in a total of 9 min of continuous walking.

Before measurement, all participants practiced treadmill walking using the BPW device, and the experimenter visually confirmed both a natural arm-swing motion and stable gait pattern before preliminary and main measurements were initiated.

Walking cadence (steps·min^−1^) was calculated by counting steps for 1 min while walking under each condition using a triaxial accelerometer. To reflect a stable walking state, the measurement interval was set to the last minute (2–3 min) of each 3 min walking bout. Estimated step length (m) was calculated by dividing walking speed (m·min^−1^) by the walking cadence (steps·min^−1^) (step length = speed·cadence^−1^). These indices were used to evaluate differences in gait pattern, including step length and rhythm, between BPW and WK.

**Figure 1:**
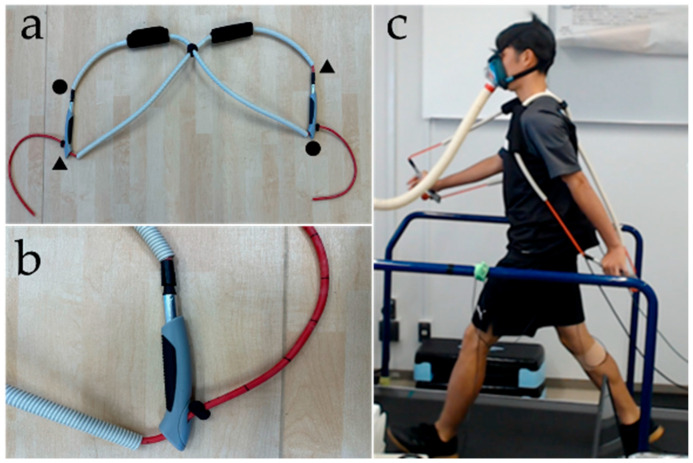
Portable device for upper-limb-resisted walking (band-pull walking [BPW] device). (**a**) Overview of the portable elastic resistance device consisting of extensible rubber tubes, a lightweight harness, and hand-held grips. The device is constructed using two extensible rubber tubes connected at positions indicated by ●–● and ▲–▲ in the figure. (**b**) Enlarged view of the adjustable attachment mechanism integrated into the grip unit, which is used to set the effective tube length and resistance. The resistance on the left and right sides can be adjusted independently. (**c**) A participant wearing the BPW device while walking on a treadmill, showing posterior and downward resistive forces applied during arm swing.

For BPW, two elastic rubber tubes with an outer diameter of 7.45 mm and thickness of 1.20 mm (TRIDEX free band tube: For-dear Rubber) were used as part of a portable device consisting of a lightweight harness and hand-held grips (Figure 1). The harness was worn on the back in a backpack-like manner, and the tubes were arranged to generate backward and downward pulling forces relative to the trunk through arm-swing movements involving shoulder and elbow extension. This configuration was designed to apply backward resistance during larger arm-swing movements. To ensure standardization of device setup, the harness was positioned so that the upper margin aligned with the inferior angle of the scapula, and the tube attachment points were symmetrically arranged relative to the trunk. Grip orientation was maintained in a neutral forearm position. Before each trial, the experimenter visually confirmed proper harness fit, tube alignment, and grip position to minimize setup variability.

The tube load was individually standardized based on the MVC of the long head of the TB to minimize the variability in external load associated with differences in body size and upper-limb length among participants, while providing a consistent load to the upper-limb muscles without interfering with arm-swing movements during walking. A new elastic tube was used for each participant, which was not reused across participants. To minimize cumulative material fatigue effects, each tube was used in only one experimental session.

The MVC was measured with the participants seated on a chair without a backrest, with the shoulder joint fixed at 0° and the elbow joint fully extended. A resistance pad attached to the distal part of the wrist was connected to a load cell, and the participants exerted maximal isometric effort in the direction of elbow extension for 5 s. Three trials were performed for each participant, with 60 s of rest between trials. The mean value of EMG was calculated during the 2–4 s interval for each trial, and the average of the three trials was defined as the MVC of the participant.

For all participants, forearm length was normalized by defining the radial styloid process as 0% and the olecranon as 100%, and tube length was set at five levels (0%, 25%, 50%, 75%, and 100%) based on this proportion. BPW was performed under each tube-length condition, and the muscle activity of the long head of the TB (%MVC) was recorded. A linear regression model used for each participant to estimate the relationship between tube length and TB muscle activity (%MVC) revealed a strong linear relationship (R^2^ = 0.92), confirming that linear approximation was appropriate within the range of tube elongation used in this study. Based on this regression equation, the tube length corresponding to a TB muscle activity level of 8% MVC was calculated, and tube load was individually set for each participant.

This muscle activity level falls within the range generally observed during activities of daily living and light aerobic exercise (approximately 5–15%MVC), and has been reported to represent an intensity that does not induce excessive fatigue or muscle blood flow restriction [33]. Furthermore, a preliminary experiment confirmed that a load near 8% MVC neither visually interfere with arm-swing movements during walking nor induce changes in gait pattern, and provided a clear sensation of external resistance to the upper limbs. Consequently, in this study, the tube length corresponding to approximately 8% MVC of TB activity was individually set based on the regression equation, and the main experiment was conducted. Importantly, the purpose of the present study was not to determine the optimal resistance intensity but to establish a standardized and reproducible loading condition under controlled laboratory settings. Therefore, 8% MVC should be interpreted as a practical standardization criterion rather than an optimal or maximal loading level.

To characterize the force–elongation relationship of the rubber tube, a test was conducted in which one end of the tube was fixed, and the other end was connected to a load cell and statically elongated. Elongation (x) was defined as the distance (cm) from the natural length, and tension (y) was defined as the force (kgf) measured by the load cell. Before measurement, several pre-stretching cycles were performed within the designated range to reduce the influence of the history-dependent properties of the material. The elastic tube used in this study generated approximately 1.2 kgf, 2.1 kgf, and 2.6 kgf at elongations of 5, 15, and 25 cm, respectively. Within this range, the tension–elongation relationship was approximately linear (R^2^ = 0.99). The mean estimated tube tension was 2.1 ± 0.4 kgf in men and 1.0 ± 0.1 kgf in women. These values represent reference values indicating the magnitude of external resistance generated when TB activity was set at approximately 8% MVC in this study, and the estimated tension serves as a supplementary value describing device characteristics.

### 2.3. Experimental Procedures

Participants arrived at the laboratory at least 30 min before the start of the experiment, and their height, body mass, and resting blood pressure were measured to confirm their health status. EMG electrodes (EMG amplifier SX230-1000, Biometrics Ltd., Newport, UK) were attached to the target muscles, and a triaxial accelerometer (MicroStone Co., Ltd., Nagano, Japan) was attached to the lateral side of the left knee joint to measure gait cycles and step counts at each speed. The walking cadence (steps·min^−1^) was calculated from the number of steps recorded for 1 min during the last minute (2–3 min) of each 3 min walking bout, when walking was stable, to reflect the steady-state walking rhythm.

After attaching the EMG electrodes and triaxial accelerometer, a face mask was fitted for expired gas analysis. Following a seated familiarization period until breathing stabilized, a 5 min seated resting measurement was noted to record resting metabolic rate and resting EMG. For safety monitoring during the experiment, ECG waveforms were monitored using a patient monitor (BSM-2401, Nihon Kohden Corporation, Tokyo, Japan).

After the resting measurement, BPW and WK were performed in a randomized order. Walking was conducted on a treadmill with a 3% incline, with a stepwise increase in speed from 60 m·min^−1^ (3 min) to 80 m·min^−1^ (3 min) and then to 100 m·min^−1^ (3 min), resulting in a total of 9 min of continuous walking. RPE was assessed using the OMNI scale [34].

Between conditions, sufficient seated rest was provided until the HR returned to resting levels to minimize the effects of muscle fatigue and cardiorespiratory fatigue.

### 2.4. Measurements

#### 2.4.1. Muscle Activity

To evaluate muscle activity during walking, surface EMG was recorded from the left upper-limb muscles (TB, BB, and DM) and right lower-limb muscles (VL and GL) [32,35,36]. After skin abrasion and degreasing, electrodes were attached at the designated locations.

EMG signals were sampled at 1 kHz and acquired using an analog-to-digital (A/D) converter (PowerLab 16/30, ADInstruments Pty Ltd., Sydney, Australia) and transferred to a personal computer. Data were analyzed using biological signal analysis software (LabChart Pro 7.3.7, ADInstruments Pty Ltd., Sydney, Australia). The EMG signals were band-pass filtered at 20–450 Hz, full-wave rectified, and processed using a root mean square (RMS) algorithm (RMS window: 100 ms) to quantify muscle activity.

Gait cycles were synchronized with signals from a triaxial accelerometer signal (MicroStone Co., Ltd., Nagano, Japan) attached to the lateral side of the left knee joint. Ten consecutive gait cycles were extracted from stable walking periods under each condition, and the mean RMS value was used as the representative value for each condition [37,38]. Before analysis, the raw waveforms of each trial were visually inspected to confirm the absence of obvious movement disturbances or artifacts.

For comparisons among conditions of muscle activity, the maximum RMS value obtained in all conditions (WK and BPW at each speed) for each participant was defined as 100% (peak) for each muscle [39,40]. RMS values for each condition (WK60, BPW60, WK80, BPW80, WK100, and BPW100) were normalized to this peak and expressed as percentage of dynamic peak (%dynamic peak) [41,42]. This normalization enabled comparison of relative changes in muscle activity within participants without using condition-dependent reference values [39,43]. Although %MVC of the TB was used to standardize external resistance across participants, EMG outcomes were normalized to %dynamic peak for inter-condition comparisons during walking. MVC-based normalization reflects maximal voluntary capacity under static conditions, whereas dynamic peak normalization captures task-specific activation patterns during cyclic locomotor activities [44]. Thus, %MVC was adopted for load prescription, while %dynamic peak was selected to enhance intra-subject comparability and robustness under dynamic walking conditions.

#### 2.4.2. Oxygen Uptake and Heart Rate

$\overset{・}{V}$O_2_ was measured using a metabolic system (AE-310S, Minato Medical Science Co., Ltd., Osaka, Japan). Prior to each experiment, the flow sensor was calibrated using a 2 L syringe, and the O_2_ and CO_2_ sensors were calibrated using certified standard gases of known concentrations (O_2_: 20.72% in N_2_ balance; O_2_: 15.09% and CO_2_: 5.00% in N_2_ balance; Sumitomo Seika Chemicals Co., Ltd., Osaka, Japan). Expired gas was analyzed in the expire mode.

HR was obtained from the ECG analog signal output from a patient monitor (BSM-2401, Nihon Kohden Corporation, Tokyo, Japan), which was input to a data acquisition system (PowerLab 16/30, ADInstruments Pty Ltd., Sydney, Australia) and A/D converted at 1 kHz. The signal was transferred to a personal computer and continuously recorded using biological signal analysis software (LabChart Pro 7.3.7, ADInstruments Pty Ltd., Sydney, Australia). R waves were automatically detected using the peak detection algorithm in LabChart, and HR was calculated from each R–R interval.

For each exercise condition, $\overset{・}{V}$O_2_ and HR were analyzed over the steady-state period from 2 to 3 min after the start of the exercise, and the mean value over this interval was used as the representative value. As an index of relative exercise intensity, METs were calculated by defining 1 MET as 3.5 mL·kg^−1^·min^−1^, and relative $\overset{・}{V}$O_2_ values expressed as METs were compared in all conditions. Cardiorespiratory RPE was assessed using the OMNI scale immediately after completion of each 3 min speed stage.

#### 2.4.3. Rating of Perceived Exertion

RPE was assessed using the OMNI scale [34] for three components—upper limbs (UL-RPE), lower limbs (LL-RPE), and cardiorespiratory (CR-RPE)—immediately after completion of each speed stage (3 min).

#### 2.4.4. Walking Cadence and Estimated Step Length

The walking cadence (steps·min^−1^) and estimated step length were calculated using the triaxial accelerometer data and walking speed. They were used as indices to evaluate the gait pattern, including step length and rhythm under each condition.

### 2.5. Statistical Analysis

All variables are presented as means ± standard deviations. To examine the effects of walking condition (WK and BPW) and speed (60, 80, and 100 m·min^−1^), METs, HR, muscle activity (%dynamic peak), RPE, walking cadence, and estimated step length were analyzed using a two-way repeated-measures analysis of variance with condition (two levels) and speed (three levels) as factors. Sphericity was assessed using Mauchly’s test, and violation of the sphericity assumption prompted application of the Greenhouse–Geisser correction. Significant main effects or interactions led to post hoc comparisons using the Bonferroni correction to examine simple effects of condition (WK vs. BPW) at each speed based on estimated marginal means (EMMs). Partial η^2^ was calculated as the effect size for each main effect and interaction. No missing values were observed, and no outliers were excluded from any analysis. Statistical analyses were performed using SPSS Statistics (version 22.0 for Windows; IBM Corp., Armonk, NY, USA ), and the statistical significance was set at *p* < 0.05.

### 2.6. Ethics

This study was conducted with the approval of the Ethics Committee for Research Involving Human Subjects at Shizuoka University (approval number: 20-17).

## 3. Results

### 3.1. Muscle Activity Responses

#### 3.1.1. TB

For TB muscle activity (%dynamic peak), the main effect of condition (WK vs. BPW) was significant [F(1, 13) = 168.79, *p* < 0.001, partial η^2^ = 0.928], with BPW showing a markedly higher muscle activity than WK.

**Figure 2:**
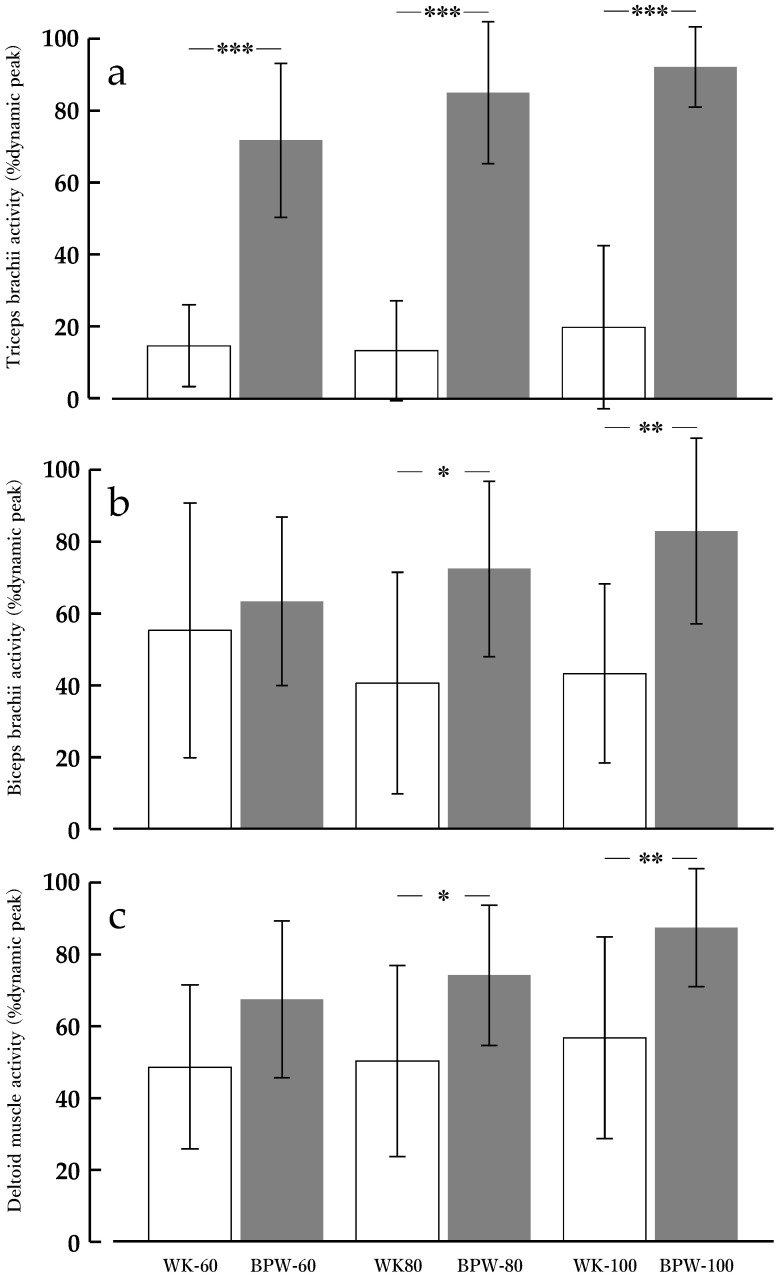
Upper-limb muscle activity during normal walking (WK) and band-pull walking (BPW) at 60, 80, and 100 m·min^−1^. Electromyography amplitude is expressed as percentage of dynamic peak. Values are presented as means ± standard deviations (*n* = 14). (**a**) Triceps brachii (TB), (**b**) biceps brachii (BB), and (**c**) deltoid muscle (DM). * *p* < 0.05, ** *p* < 0.01, *** *p* < 0.001 for BPW vs. WK at the same speed (Bonferroni-adjusted).

The main effect of speed was also significant [F(2, 26) = 6.90, *p* = 0.004, partial η^2^ = 0.347], indicating an overall increase in muscle activity with increasing walking speed. In contrast, the condition × speed interaction was not significant [F(2, 26) = 1.70, *p* = 0.203, partial η^2^ = 0.115] (Figure 2a).

#### 3.1.2. BB

For BB muscle activity (%dynamic peak), the main effect of condition (WK vs. BPW) was significant [F(1, 13) = 4.96, *p* = 0.044, partial η^2^ = 0.276], with BPW showing a higher muscle activity than WK (EMMs: WK 46.2 ± 6.9, BPW 72.8 ± 5.7%dynamic peak).

In contrast, the main effect of speed did not reach statistical significance [F(1.38, 17.98) = 0.88, *p* = 0.394 (Greenhouse–Geisser corrected), partial η^2^ = 0.064].

The condition × speed interaction reached statistical significance [F(2, 26) = 6.60, *p* = 0.005, partial η^2^ = 0.337], indicating speed-dependent variation in the magnitude of the condition difference.

Examination of simple effects of condition using Bonferroni correction showed a significantly higher BB muscle activity during BPW than during WK at 80 m·min^−1^ (*p* < 0.05) and 100 m·min^−1^ (*p* < 0.01), whereas no statistically significant inter-condition difference emerged at 60 m·min^−1^ (Figure 2b).

#### 3.1.3. DM

For DM activity (%dynamic peak), the main effect of condition (WK vs. BPW) was significant [F(1, 13) = 11.19, *p* = 0.005, partial η^2^ = 0.463], with BPW showing a higher muscle activity than WK (EMMs: WK 51.8 ± 6.1, BPW 76.3 ± 3.0%dynamic peak).

The main effect of speed was also significant [F(2, 26) = 5.46, *p* = 0.010, partial η^2^ = 0.296], indicating increased muscle activity with increasing walking speed. In contrast, the condition × speed interaction did not reach statistical significance [F(2, 26) = 0.71, *p* = 0.501, partial η^2^ = 0.052], and the pattern of increase across speeds remained similar between conditions (Figure 2c).

#### 3.1.4. VL

**Figure 3:**
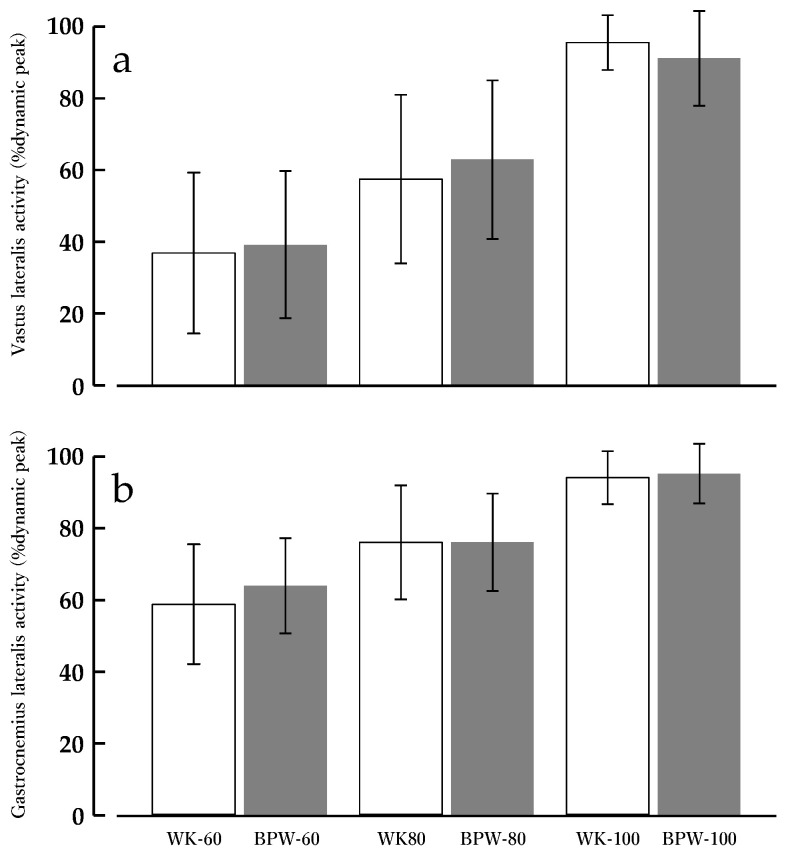
Lower-limb muscle activity during normal walking (WK) and band-pull walking (BPW) at 60, 80, and 100 m·min^−1^. Electromyography amplitude is expressed as percentage of dynamic peak. Values are presented as means ± standard deviations (*n* = 14). (**a**) Vastus lateralis (VL) and (**b**) gastrocnemius lateralis (GL).

For VL muscle activity (%dynamic peak), the main effect of condition (WK vs. BPW) was not significant [F(1, 13) = 0.16, *p* = 0.694, partial η^2^ = 0.012]. In contrast, the main effect of speed reached statistical significance [F(2, 26) = 47.47, *p* < 0.001, partial η^2^ = 0.785], indicating a substantial increase in muscle activity with increasing walking speed. The condition × speed interaction was not significant [F(2, 26) = 2.12, *p* = 0.140, partial η^2^ = 0.140] (Figure 3a).

#### 3.1.5. GL

For GL muscle activity (%dynamic peak), the main effect of condition (WK vs. BPW) was not significant [F(1, 13) = 0.62, *p* = 0.445, partial η^2^ = 0.046]. In contrast, the main effect of speed was significant [F(2, 26) = 49.31, *p* < 0.001, partial η^2^ = 0.791], indicating increased muscle activity with increasing walking speed. The condition × speed interaction was not significant [F(2, 26) = 1.46, *p* = 0.250, partial η^2^ = 0.101] (Figure 3b).

### 3.2. METs

For METs, the main effect of condition was significant [F(1, 13) = 36.014, *p* < 0.001, ηp^2^ = 0.735]. The main effect of speed was also significant [F(2, 26) = 362.706, *p* < 0.001, ηp^2^ = 0.965], with METs increasing stepwise with higher walking speeds. Additionally, the condition × speed interaction was significant [F(1.648, 21.418) = 4.047, *p* = 0.039, ηp^2^ = 0.237].

**Figure 4:**
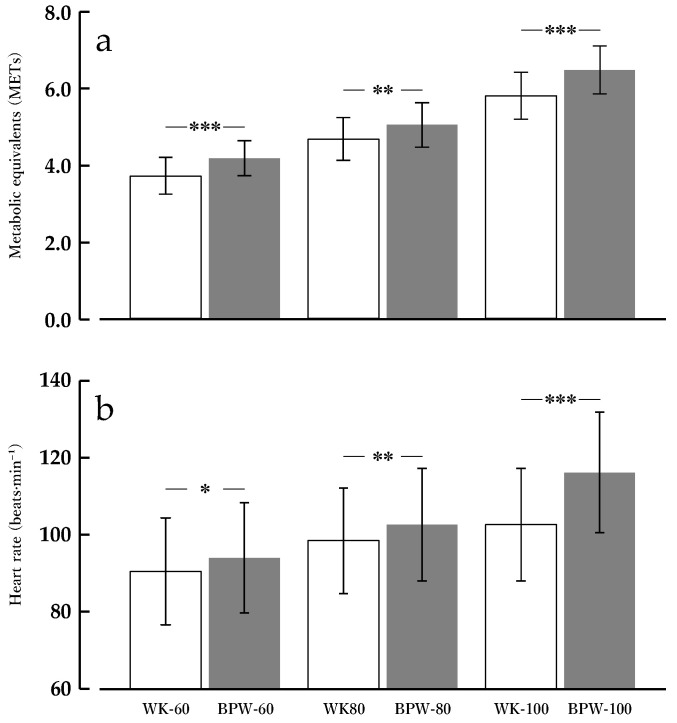
METs and Heart rate during normal walking (WK) and band-pull walking (BPW) at 60, 80, and 100 m·min^−1^. Values are presented as means ± standard deviations (*n* = 14). (**a**) Metabolic equivalents (METs) and (**b**) heart rate (HR). * *p* < 0.05, ** *p* < 0.01, *** *p* < 0.001 for BPW vs. WK at the same speed (Bonferroni-adjusted).

Post hoc comparisons using Bonferroni correction showed that at 60 m·min^−1^, the MET value for BPW (4.2 ± 0.4 METs) was significantly higher than that for WK (3.7 ± 0.5 METs, *p* < 0.001). At 80 m·min^−1^, the MET value for BPW (5.0 ± 0.6 METs) exceeded that for WK (4.7 ± 0.6 METs, *p* = 0.003). At 100 m·min^−1^, the MET value for BPW (6.5 ± 0.6 METs) remained significantly higher than that for WK (5.8 ± 0.6 METs, *p* < 0.001) (Figure 4a).

### 3.3. HR

For HR, the main effect of condition was significant [F(1, 13) = 22.150, *p* < 0.001, ηp^2^ = 0.630]. The main effect of speed was also significant [F(2, 26) = 473.733, *p* < 0.001, ηp^2^ = 0.973], indicating that HR increased with increasing speed. Additionally, the condition × speed interaction was significant [F(2, 26) = 5.738, *p* = 0.009, ηp^2^ = 0.306].

Post hoc comparisons using Bonferroni correction showed that at 60 m·min^−1^, HR during BPW (94.0 ± 14.3 bpm) was significantly higher than that during WK (90.5 ± 13.8 bpm, *p* < 0.05). At 80 m·min^−1^, HR during BPW (102.6 ± 14.6 bpm) exceeded that during WK (98.4 ± 13.6 bpm, *p* < 0.01). At 100 m·min^−1^, the difference further increased, with HR during BPW (116.1 ± 15.6 bpm) remaining significantly higher than that during WK (108.6 ± 14.0 bpm, *p* < 0.001) (Figure 4b).

### 3.4. RPE

#### 3.4.1. UL

For UL-RPE, the main effect of condition (BPW vs. WK) was significant [F(1, 13) = 33.652, *p* < 0.001, ηp^2^ = 0.721]. The main effect of speed was also significant [F(2, 26) = 27.019, *p* < 0.001, ηp^2^ = 0.675], with RPE increasing at higher walking speeds. Additionally, the condition × speed interaction was significant [F(2, 26) = 10.920, *p* < 0.001, ηp^2^ = 0.457].

**Figure 5:**
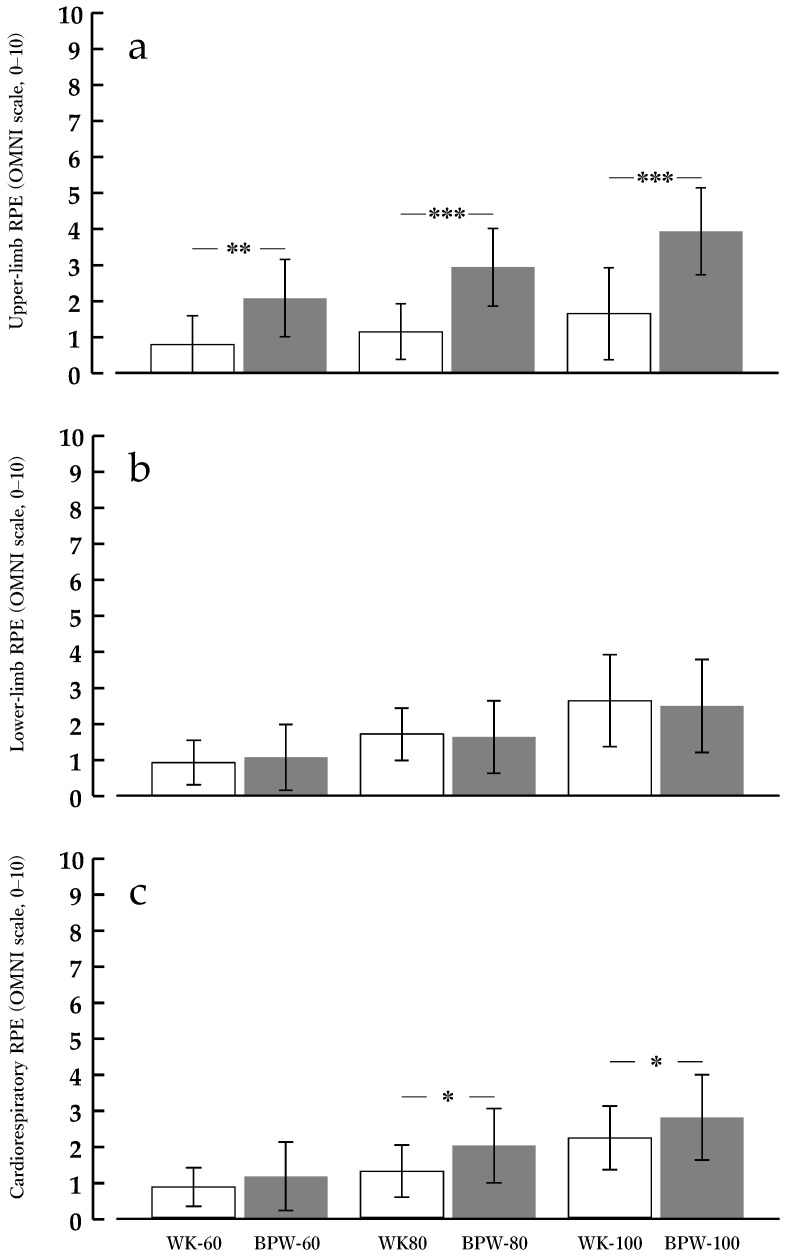
Rating of perceived exertion during normal walking (WK) and band-pull walking (BPW) at 60, 80, and 100 m·min^−1^. RPE was assessed using the OMNI scale (0–10). Values are presented as means ± standard deviations (*n* = 14). (**a**) Upper-limb RPE, (**b**) lower-limb RPE, and (**c**) cardiorespiratory RPE. * *p* < 0.05, ** *p* < 0.01, *** *p* < 0.001 for BPW vs. WK at the same speed (Bonferroni-adjusted).

Post hoc comparisons using Bonferroni correction showed that at 60 m·min^−1^, RPE for BPW (2.1 ± 1.1) was significantly higher than that for WK (0.8 ± 0.8, *p* < 0.01). At 80 m·min^−1^, RPE for BPW (2.9 ± 1.1) exceeded that for WK (1.1 ± 0.8, *p* < 0.001). At 100 m·min^−1^, the difference further increased, with RPE for BPW (3.9 ± 1.2) remaining significantly higher than that for WK (1.6 ± 1.3, *p* < 0.001) (Figure 5a).

#### 3.4.2. LL

For LL-RPE, the main effect of condition (BPW vs. WK) was not significant [F(1, 13) = 0.013, *p* = 0.909, ηp^2^ = 0.001]. The condition × speed interaction was also not significant [F(2, 26) = 1.090, *p* = 0.351, ηp^2^ = 0.077]. In contrast, the main effect of speed was significant [F(2, 26) = 20.056, *p* < 0.001, ηp^2^ = 0.607], with LL-RPE increasing stepwise with higher walking speeds (Figure 5b).

#### 3.4.3. CR

For CR-RPE, the main effect of condition was significant [F(1, 13) = 10.848, *p* = 0.006, ηp^2^ = 0.455], with BPW showing significantly higher values than WK.

The main effect of speed was also significant [F(2, 26) = 27.573, *p* < 0.001, ηp^2^ = 0.680], indicating stepwise increases in cardiorespiratory RPE with higher walking speeds. In contrast, the condition × speed interaction was not significant [F(2, 26) = 1.857, *p* = 0.176, ηp^2^ = 0.125], suggesting that the BPW-related increase in CR-RPE was consistent across all speed levels and not speed-dependent (Figure 5c).

### 3.5. Cadence

For cadence (steps·min^−1^), the main effect of condition was significant [F(1, 13) = 5.21, *p* = 0.040, partial η^2^ = 0.286], with BPW showing overall lower cadence than WK. The main effect of speed was also significant [F(2, 26) = 174.74, *p* < 0.001, partial η^2^ = 0.931], indicating that cadence increased with higher walking speeds. In contrast, the condition × speed interaction was not significant [F(2, 26) = 0.04, *p* = 0.959], suggesting that the cadence difference between BPW and WK remained consistent across all speeds.

**Figure 6:**
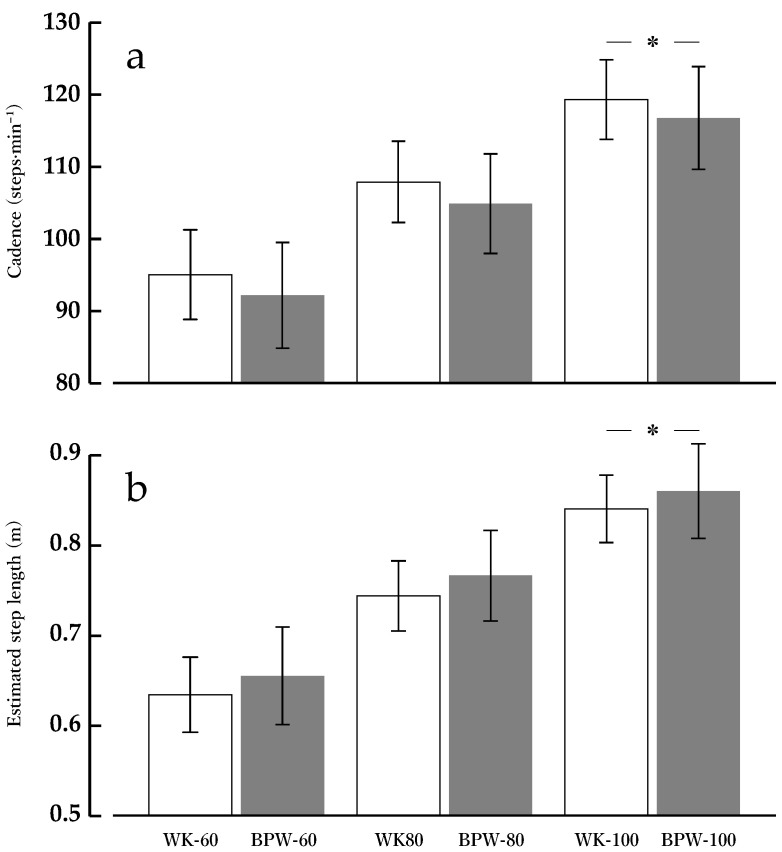
Gait parameters during normal walking (WK) and band-pull walking (BPW) at 60, 80, and 100 m·min^−1^. (**a**) Cadence (steps·min^−1^) and (**b**) estimated step length (m). Values are presented as means ± standard deviations (*n* = 14). Step length was estimated as walking speed divided by cadence. * *p* < 0.05 for BPW vs. WK at the same speed (Bonferroni-adjusted).

Based on EMMs, cadence was on average 2.81 steps·min^−1^ lower in BPW (104.57 ± 1.64 steps·min^−1^) than in WK (107.38 ± 1.35 steps·min^−1^, *p* = 0.040) (Figure 6a).

### 3.6. Estimated Step Length

For estimated step length, the main effect of condition was significant [F(1, 13) = 5.75, *p* = 0.032, partial η^2^ = 0.307], with BPW showing overall greater step length than WK. The main effect of speed was also significant [F(2, 26) = 239.99, *p* < 0.001, partial η^2^ = 0.949], indicating that estimated step length increased with higher walking speeds. In contrast, the condition × speed interaction was not significant [F(2, 26) = 0.026, *p* = 0.975], indicating that the step length increase with BPW was consistent across all speeds.

Based on EMMs, the estimated step length was on average 0.021 m greater in BPW (0.760 ± 0.012 m) than in WK (0.739 ± 0.009 m, *p* = 0.032) (Figure 6b).

## 4. Discussion

The purpose of this exploratory study was to examine the effects of BPW using a portable elastic tube device on cardiorespiratory and neuromuscular responses in healthy young adults compared with WK.

In BPW, elastic tubes generate tension during elongation, the magnitude of which varies according to the degree of elongation [24,25]. Due to this mechanical property, resistive loads that vary over time are applied to the upper limbs during arm swing. In this study, the TB showed significantly higher muscle activity during BPW than during WK at all walking speeds, with a significant main effect of conditions. The TB has been reported as one of the primary muscles involved in elbow extension [45], and the observed increase in TB activity during BPW suggests greater neuromuscular demands due to the addition of tube resistance or associated changes in arm swing.

The BB represents an upper-limb muscle involved in elbow flexion [46]. In this study, BB activity differed between conditions according to walking speed. BPW showed a significantly higher BB activity than WK at 80 and 100 m·min^−1^, whereas no significant differences were observed at 60 m·min^−1^. This finding suggests that, under conditions in which arm-swing increases with increasing walking speed [47], upper-limb muscle recruitment may have become more pronounced.

Furthermore, the DM contributes to arm-swing, including shoulder flexion and extension [48]. The increased DM activity observed during BPW suggests increased neuromuscular demands on the shoulder musculature due to tube resistance or changes in upper-limb movement patterns. Because no condition × speed interaction was observed, the increase in DM activity was not limited to a specific speed range and may have occurred as an additive effect on all walking speeds.

Although VL and GL showed increased muscle activity with increasing walking speed, no significant condition-related differences were observed between BPW and WK. In contrast, BPW showed a decrease in cadence and an increase in estimated step length compared with WK, with significant differences in both variables, particularly at 100 m·min^−1^.

One possible explanation for these gait changes involves the application of varying external resistance to the upper limbs over time during arm swing in BPW. Such resistance may alter the amplitude and timing of arm movements and, through upper–lower limb coordination, influence cadence. Eke-Okoro et al. (1997) [47] and Ford et al. (2007) [49] reported that manipulating arm-swing conditions altered gait patterns and that changes in upper–lower limb coordination became more apparent at higher walking speeds. Despite these observations, no condition-related differences in VL and GL activity were observed in the present study. BPW altered the cadence by −2.2 to −3.0% and estimated step length by 2.4 to 3.3% relative to WK, whereas previous studies have reported that larger manipulations of step number, such as reductions of approximately 10%, can alter lower-limb muscle activity [50]. Moreover, even under a constant walking speed, large manipulations of step length change lower-limb EMG activity [51]. Therefore, the magnitude of cadence and step length changes observed during BPW may have been insufficient to induce detectable changes in VL and GL activity. Under the speed conditions examined in this study, BPW appears consistent with a selective increase in upper-limb muscle activity while maintaining lower-limb muscle activity at levels comparable to WK. However, the lower-limb muscles evaluated in the present study were limited to the VL and GL. In general, EMG amplitude reflects neuromuscular activation but does not directly quantify mechanical loading variables such as external joint moments or joint contact forces acting on the joint [52,53]. Accordingly, the absence of EMG amplitude differences between conditions for specific muscles does not necessarily imply that joint loading remained unchanged. Therefore, caution is required when interpreting the absence of differences in lower-limb muscle activity in relation to mechanical loading at the lower-limb joints.

In the present study, BPW was associated with a reduction in cadence and an increase in the estimated step length. Although the magnitude of these changes was relatively small, they may not be negligible from a biomechanical perspective. Previous studies have demonstrated that alterations in cadence and step length can influence lower-limb loading indices, including ground reaction force patterns, joint moments, and joint contact forces [54,55]. Moreover, when gait patterns are altered, mechanical loading at the joint level may change even in the absence of marked differences in EMG amplitude [52].

Furthermore, reductions in cadence may alter impact-related variables, such as the peak vertical ground reaction force and loading rate. Likewise, increases in step length may influence joint moments by modifying the lever arm and the point of application of the ground reaction force [52,54,55]. Therefore, the data from this study cannot conclusively determine the extent to which observed gait changes influenced lower-limb joint loading.

Taken together, the present findings indicate that neuromuscular activation of the measured lower-limb muscles (VL and GL) did not increase under BPW conditions. However, these results do not directly demonstrate that mechanical loading at the lower-limb joints remained unchanged. To draw definitive conclusions about lower-limb loading, comprehensive biomechanical assessments—including ground reaction forces, joint moments, and joint contact forces—are required in future studies.

METs and HR were significantly higher during BPW than during WK at all walking speeds, indicating a consistently higher cardiorespiratory demand. Across speeds, METs increased by approximately 8–12%, whereas HR increased by approximately 4–7% compared with WK. From an exercise-prescription perspective, these findings indicate that BPW enhances exercise intensity without requiring proportional increases in walking speed, thereby potentially reducing the need for faster gait to achieve moderate-to-vigorous intensity thresholds.

In this study, no condition-related differences were observed in VL and GL muscle activity, whereas METs and HR were higher during BPW than during WK at all speeds. Previous studies have suggested that, because the upper-limb muscle mass is smaller than lower-limb muscle mass, a given amount of external work may impose a relatively higher metabolic cost on the upper-limb muscles [56,57]. Therefore, the metabolic increase observed during BPW cannot be readily explained solely by lower-limb muscle activity. Given the absence of condition-related differences in VL and GL muscle activity, the repetitive activation of the upper-limb muscles in response to external resistance may have contributed to the observed elevations in METs and HR.

The present findings, indicating that active upper-limb involvement increases energy expenditure, align with previous studies of upper-limb-involved walking modalities such as Nordic walking and dumbbell walking. During Nordic walking, energy expenditure has repeatedly been reported to exceed that observed during WK under identical walking speed conditions, with increases of approximately 10–20% depending on the condition [58,59]. During dumbbell walking, energy expenditure has been reported to be 5.8–13.0% higher than during WK [60]. These increases have been interpreted as reflecting greater mechanical work performed by the upper-limb muscles during active use, manifested as an additive metabolic response.

From a muscle activity perspective, Nordic walking has been reported to markedly increase upper-limb muscle activity, particularly the TB, DM, and BB, compared with WK [14]. In contrast, although the results for the lower-limb muscles vary depending on the muscles measured and conditions, some studies have reported less consistent increases or no significant differences [15]. This pattern of “increased upper-limb muscle activity with unchanged lower-limb muscle activity” is consistent with the present findings of BPW (increased TB, BB, and DM with unchanged VL and GL).

In this study, BPW increased METs by approximately 7.9–12.3% and HR by approximately 4.0–6.9% compared with WK, placing the magnitude of the metabolic increment within or slightly below the range reported for Nordic walking [58,59]. While the magnitude of the metabolic augmentation appears comparable, the underlying mechanisms may not be entirely identical. The metabolic increment induced by BPW may reflect not only increased upper-limb muscle activity due to external resistance but also contributions from unmeasured factors such as trunk muscle activation and postural control. Indeed, previous studies on Nordic walking have reported increased trunk muscle activity and greater demands for postural stabilization [61]. Although trunk muscle activity was not directly measured in the present study, it remains plausible that similar mechanisms contributed to the increased energy expenditure observed during BPW.

The RPE results further support these cardiorespiratory and neuromuscular findings from the perspective of subjective load. UL-RPE was significantly higher during BPW than during WK at all speeds, whereas no condition-related differences were observed in LL-RPE; consequently, CR-RPE was higher during BPW as a main effect. These RPE findings are consistent with the EMG results showing increased upper-limb muscle activity in TB, BB, and DM, along with unchanged lower-limb muscle activity in VL and GL. This pattern supports the interpretation that the metabolic increment observed during BPW was driven primarily by additional upper-limb activity. Therefore, given that cardiorespiratory responses were enhanced without increases in measured lower-limb muscle activity and LL-RPE under the present conditions, BPW may represent an upper-limb-involved walking modality capable of increasing exercise intensity primarily through increased upper-limb muscle activity.

Nordic walking and dumbbell walking can also increase energy expenditure through upper-limb involvement [58,59,60]. In Nordic walking, peak pole force and pole impulse vary with user proficiency, and these variables are associated with the magnitude of the metabolic response [16]. Moreover, changes in the poling technique (strength of propulsive pole use) can reduce the pole reaction force and metabolic responses [13], suggesting that individual technique differences in practical settings may contribute to variability in metabolic increments. In dumbbell walking, the load is carried in the upper limbs, and increased demands for postural control and trunk stabilization have been reported [19], along with changes in trunk muscle activity, hip joint moments [18], and gait pattern [17].

In contrast, BPW is characterized by the ability to apply external resistance to the upper limbs in a quantitatively and individually standardized manner through tube tension. In this study, the load was set so that TB activity approximated 8% MVC, and BPW showed consistent increases in upper-limb muscle activity and metabolic load across all speed conditions. Additionally, resistance from the elastic tubes acted throughout the entire arm-swing cycle and generated tension regardless of arm-movement direction. These mechanical properties allow relatively stable application of external resistance to the upper limbs compared with modalities such as Nordic walking, which depend strongly on ground contact timing and pole operation. Moreover, because tube tension varies with the degree of elongation, increases in arm-swing amplitude accompanying higher walking speeds may lead to greater external resistance applied to the upper limbs. Thus, BPW may theoretically function as a walking modality in which upper-limb load increases with walking speed. Arm-swing kinematics and tube elongation were not directly measured in this study; therefore, verification of this mechanism remains a focus for future research.

Although the present study demonstrated that BPW increased upper-limb muscle activity and cardiorespiratory responses compared with WK, several limitations should be acknowledged.

First, the participant sample consisted exclusively of healthy young adults. Therefore, caution is required when generalizing these findings to older adults or clinical populations, including individuals with obesity or musculoskeletal and cardiovascular diseases. Because BPW involves externally applied resistance to the upper limbs, safety and feasibility may differ in populations with shoulder or elbow impairments. Further investigation is necessary to determine the applicability and safety profile of BPW in these populations. In addition, the sample size in the present study was relatively small (*n* = 14), which may have limited the statistical power to detect small effects, particularly for lower-limb muscle activity variables. Therefore, the absence of significant condition-related differences in VL and GL activity should be interpreted with caution.

Second, aspects of the experimental design may limit the causal interpretation. The walking speed was increased in a fixed stepwise order (60 → 80 → 100 m·min^−1^) for safety. Although cardiorespiratory variables and lower-limb EMG activity increased with speed and no disproportionate elevation was observed at the final stage, a potential time-dependent or cumulative fatigue effect cannot be entirely excluded. Future studies employing randomized speed-order designs would allow a clearer separation of speed-related effects from potential fatigue or time-dependent influences.

Third, the biomechanical assessment was not comprehensive. Arm-swing kinematics and the temporal characteristics of tube elongation and tension were not directly measured. Consequently, the mechanical mechanisms linking upper-limb resistance to the observed metabolic increase remain to be clarified. Moreover, lower-limb muscle assessment was limited to the VL and GL, and joint-level mechanical variables such as ground reaction forces, joint moments, and joint contact forces were not evaluated. A more integrated biomechanical analysis incorporating upper-limb kinematics, multi-muscle EMG, and joint-mechanic variables is warranted.

Fourth, methodological considerations regarding load prescription and EMG normalization should be noted. Resistance was standardized solely on the basis of TB activity, which may not fully represent the distributed mechanical demands across the upper extremity and trunk. Moreover, individual differences in arm-swing strategy (e.g., shoulder-dominant vs. elbow-dominant movement patterns) may have influenced the distribution of mechanical load across upper-limb joints and muscles. Although TB was selected as the primary elbow extensor that directly contributes to tube elongation, variability in joint contribution cannot be completely excluded. Furthermore, muscle activity normalization relied on the dynamic peak method, which differs conceptually from MVC-based normalization when absolute muscle-load levels are considered. Future investigations should explore multi-muscle or torque-based load standardization and combined normalization strategies to provide a more comprehensive characterization of neuromuscular load.

Fifth, although device setup procedures were standardized within each experimental session and pre-stretching procedures were performed to reduce history-dependent material effects, inter-session reproducibility, inter-device variability, and long-term material stability were not formally evaluated. Therefore, the practical implementation and reproducibility of BPW outside controlled laboratory conditions require further examination. Because elastic materials may undergo changes in their mechanical properties with repeated use or over time, future studies should further investigate inter-session reproducibility, inter-device variability, and long-term material stability.

Finally, this study was limited to the assessment of acute cardiorespiratory and neuromuscular responses. The long-term training adaptations to BPW, including potential improvements in aerobic capacity, upper-limb strength, and body composition, remain unknown. Therefore, longitudinal intervention studies are particularly important to determine whether regular BPW training can lead to meaningful physiological adaptations and contribute to exercise prescription and health promotion.

Based on these considerations, future research should further elucidate the physiological and biomechanical mechanisms underlying BPW. Specifically, (1) studies in older and clinical populations are required to establish safety, feasibility, and practical applicability; (2) experimental designs employing randomized speed orders would strengthen causal inference by more clearly separating speed-related and time-dependent effects; (3) comprehensive biomechanical analyses incorporating upper-limb kinematics, multi-muscle EMG, joint moments, and ground reaction forces are needed to clarify the mechanical mechanisms underlying the observed metabolic increment and potential variability in movement strategies; (4) alternative load-standardization approaches, including multi-muscle or torque-based methods combined with refined normalization strategies, should be explored to better characterize neuromuscular load; and (5) investigations of reproducibility, real-world implementation, and long-term training adaptations are essential to establish the value of BPW for exercise prescription. Collectively, these efforts will help define the mechanistic basis, safety profile, and practical significance of BPW as a novel upper-limb resisted walking modality.

## 5. Conclusions

This study examined the effects of BPW using a portable elastic tube device on cardiorespiratory responses and muscle activity compared with WK. BPW significantly increased activity in the measured upper-limb muscles, including the TB, BB, and DM, under all conditions of walking speed, whereas no significant changes in VL and GL muscle activity were observed. Correspondingly, BPW consistently increased METs and HR, resulting in higher cardiorespiratory demand than WK. Additionally, UL-RPE and cardiorespiratory RPE increased, whereas LL-RPE remained comparable to those observed during WK. Within the conditions tested in healthy young adults, these findings suggest that BPW increases cardiorespiratory responses primarily in association with increased activation of the upper-limb muscles, without increases in the measured lower-limb muscle activity or LL-RPE.

However, the present findings are limited to acute responses in healthy young adults, and caution is required when generalizing these results to other age groups or clinical populations. Furthermore, lower-limb joint-level mechanical loading was not directly assessed. More studies are needed to elucidate the biomechanical mechanisms underlying the observed metabolic increase and to evaluate the safety, applicability to broader populations, and long-term training adaptations of BPW.

## Figures and Tables

**Table 1:** Physical characteristics of the participants.

Variable	Total (*n* = 14)	Men (*n* = 9)	Women (*n* = 5)
Age (years)	22.4 ± 2.0	22.9 ± 2.3	21.2 ± 1.1
Height (cm)	169.4 ± 7.6	172.2 ± 5.4	161.5 ± 5.7
Body mass (kg)	64.9 ± 14.0	70.4 ± 10.7	51.3 ± 7.0
Body fat (%)	14.7 ± 6.3	12.4 ± 4.1	18.8 ± 6.3

Values are presented as means ± standard deviations (SDs).

## Data Availability

The data are available on reasonable request from the corresponding author.

## Abbreviations

The following abbreviations are used in this manuscript:

BPW	band-pull walking
WK	normal walking
TB	triceps brachii muscle
BB	biceps brachii muscle
DM	deltoid muscle
VL	vastus lateralis muscle
GL	gastrocnemius lateralis muscle
EMG	electromyography
MVC	maximum voluntary contraction
$\overset{・}{V}$O_2_	oxygen uptake
HR	heart rate
RPE	rating of perceived exertion
METs	metabolic equivalents
UL-RPE	Upper-limb rating of perceived exertion
LL-RPE	Lower-limb rating of perceived exertion
CR-RPE	Cardiorespiratory rating of perceived exertion
